# EPYC functions as a novel prognostic biomarker for pancreatic cancer

**DOI:** 10.1038/s41598-024-51478-w

**Published:** 2024-01-06

**Authors:** Zhen Yang, Honglin Li, Jie Hao, Hanwei Mei, Minghan Qiu, Huaqing Wang, Ming Gao

**Affiliations:** 1 Department of Clinical Laboratory, Tianjin Union Medical Center of Nankai University, Tianjin, China; 2 The Institute of Translational Medicine, Tianjin Union Medical Center of Nankai University, Tianjin, China; 3 Department of Clinical Laboratory, Dachuan District People’s Hospital, Sichuan, China; 4 Department of Thyroid and Breast Surgery, Tianjin Key Laboratory of General Surgery in Construction, Tianjin Union Medical Center of Nankai University, Tianjin, China; 5 Department of Oncology, Tianjin Union Medical Center of Nankai University, Tianjin, China

**Keywords:** Cancer, Cell biology, Computational biology and bioinformatics, Molecular biology, Oncology

## Abstract

Pancreatic cancer (PC) has become a worldwide challenge attributed to its difficult early diagnosis and rapid progression. Treatments continue to be limited besides surgical resection. Hence, we aimed to discover novel biological signatures as clinically effective therapeutic targets for PC via the mining of public tumor databases. We found that epiphycan (EPYC) could function as an independent risk factor to predict the poor prognosis in PC based on integrated bioinformatics analysis. We downloaded associated PC data profiles from The Cancer Genome Atlas (TCGA) and Gene Expression Omnibus (GEO) online websites, then applied the software Rstudio to filter out genes under the strict criteria. After the batch survival analysis using Log-rank test and univariate cox regression, we obtained 39 candidate genes. Subsequently, we narrowed the scope to 8 genes by establishing a Lasso regression model. Eventually, we focused on 2 genes (EPYC and MET) by further building a multivariate cox regression model. Given that the role of EPYC in PC remains obscure, we then performed a series of molecular functional experiments, including RT-qPCR, CCK8, EdU, colony formation, Transwell, western blot, cell live-dead staining, subcutaneous tumor formation, to enhance our insight into its underlying molecular mechanisms. The above results demonstrated that EPYC was highly expressed in PC cell lines and could promote the proliferation of PCs via PI3K-AKT signaling pathway in vivo and in vitro. We arrived at a conclusion that EPYC was expected to be a biological neo-biomarker for PC followed by being a potential therapeutic target.

## Introduction

Pancreatic cancer (PC) is one of the most common malignant gastrointestinal tumors in the world today, and it is known for its high malignancy and poor prognosis^1–3^. Clinical treatments for PC are limited and ineffective^4^, therefore it is particularly important to explore novel and effective therapeutic strategies to reduce the mortality of PC patients. Unfortunately, the underlying molecular mechanisms of its occurrence and development are still poorly understood. Hence, deeper exploration for more instructive biological targets of PC followed by elucidating the underlying molecular mechanism would help enrich therapeutic tools and thus improve the survival rate of PC patients.

Bioinformatics is a new discipline formed with the rapid development of life science and computer science^5,6^. With the construction of tumor-related databases worldwide, the application of bioinformatics to mine useful information in tumors combined with experimental validation has gradually become a neoteric and powerful tool for the tumor research. For instance, Liu et al. identified and confirmed 3 new-found LncRNA prognostic biomarkers for colorectal cancer using the above method^7^. Chen et al. also driven the identification and validation of several hub genes for bladder cancer via relevant online databases^8^. In addition, Han et al. reported a novel tumor stemness-associated ceRNA axis in lung adenocarcinoma based on the bioinformatics analysis combined with the validation of molecular assays^9^. In fact, there are many other similar studies up to now^10–12^, which indicates that this method is an effective tool for oncology research. Currently, the two most widely used tumor-related databases in the world are TCGA and GEO. Therefore, we combined the PC data from these two databases in this work and finally identified EPYC as a potential novel therapeutic target for PC via rigorous analysis and experimental validation.

Epiphycan (EPYC), alias dermatan sulfate proteoglycan 3 (DSPG3), is a member of a small family of leucine-rich repetitive proteoglycans^13^. This gene located on human chromosome 12 is a protein-coding gene that consists of seven exons. And it can regulate the fibril formation by interacting with collagen fibrils and other extracellular matrix proteins^14^. EPYC has been found to function crucial roles in a variety of human diseases, such as osteoarthritis^15,16^, high myopia^17,18^, rheumatoid arthritis^19^, and so on. Nonetheless it is not clear enough that what roles EPYC plays in tumors. EPYC has recently been reported to perform vital parts as an independent risk factor in ovarian cancer^20^ and prostate cancer^21^. However, there are no related studies reported about the role of EPYC in PC. In this work, we finally found that EPYC could promote the malignant regression of PC and could be regarded as an independent risk factor to predict poor outcomes, which provided a new idea for the clinical treatment of PC.

## Materials and methods

### Data acquisition

The whole public data in this work can be found in TCGA (https://portal.gdc.cancer.gov/) and GEO (https://www.ncbi.nlm.nih.gov/gds/?term =) online database. The software RStudio (Version 4.1.3) was applied for the preliminary processing including data download, filtering, sorting, etc. We filtered out genes with zero expression in the whole samples. All the source data including the running codes involved in this study for bioinformatics analysis could be accessed at the following link: https://www.jianguoyun.com/p/DSHwSXMQ-sCrCxiH1p8FIAA. The Human Protein Atlas (HPA, https://www.proteinatlas.org/) database was used to analyze the protein levels of EPYC through immumohistochemical staining. The GEPIA (http://gepia.cancer-pku.cn/detail.php) database was used to analyze the correlation between EYPC expression level and PI3K signaling pathway.

### Differential expression analysis and functional enrichment analysis

In view of the different data formats, the differential expression analysis of TCGA data and GEO data were executed in different ways. We used three different R packages (“Limma”, “edgeR”, “DESeq2”) to perform differential expression analysis on the TCGA data separately, and then took the intersection. As for GEO data, we applied only “Limma” R package. Nonetheless, our criteria for screening differentially expressed genes (DEGs) was consistent (*p* < 0.05, FC > 1). Then related R packages (“pheatmap”, “ggplot2”, “dplyr”) were used to draw heatmaps and volcano plots to visualize the whole DEGs that were screened previously. In addition, Gene Ontology (GO) and Kyoto Encyclopedia of Genes Genomes (KEGG) functional enrichment analysis of all the candidate DEGs were based on the DAVID online database (https://david.ncifcrf.gov/home.jsp).

### The batch survival analysis (log-rank test and univariate cox regression)

The optimal node was set according to the expression level of each gene in samples. Then all the samples were divided into two groups (high and low) based on the above node, and the batch survival analysis was performed circularly using two R packages (“survival”, “survminer”). Kaplan Meier survival curves and Cox proportional hazard ratio (HR) were plotted for top-selected genes using SPSS version 20 and 95% CI. All the data were analyzed under screening criteria, including the cut-off threshold of HR > 1 and *P* < 0.05. Finally, the statistically significant (*p* < 0.05) genes were calculated out by running corresponding codes.

### The construction and validation of relevant models (Lasso and Multivariate cox regression)

All the TCGA samples were divided into training group and test group using “caret” R package. The training group data was applied for subsequent model establishment. The test group data and GEO data were both used to verify the credibility of the model. The input data for constructing a lasso regression model was the expression matrix of tumor samples with the actual survival state. Three factors were involved in the construction process, namely degrees of freedom, residual ratio, and model parameters. The main R package was “glmnet”. The input data for constructing the multivariate cox regression proportional hazards regression model were the expression matrix of finally candidate genes and their corresponding clinical information. The main application function involved was survival: coxph. As for the validation of models, we organized the validation data into a format consistent with the input data. The results were then visualized mainly based on whether the actual survival status of patients was close to the predicted survival status and whether the difference in survival between the high and low risk groups was significant.

### Cell culture and cell count

The two PCs (PANC-1, CFPAC-1) and the nonmalignant pancreatic ductal epithelial cell line HPDE6-C7 were both obtained from hepatobiliary surgery laboratory of Army Military Medical University. All the cells were cultured in high-glucose compete DMEM medium supplemented with 10% FBS (Gibco, USA) in a constant humidity incubator (37 °C, 5% CO_2_). The 3 × 10^5^ cells were seeded in 6-well plates and incubated for 48 h after transfection with related plasmids. Then all the cells of each plate were counted after trypsinization, 10 mL of cell suspension and 10 mL trypan blue were fully mixed followed by performing cell count via TC20 automated cell counter (Bio-Rad, USA).

### Knockdown of EPYC in cancer cells

The lentiviral interference plasmids (LV-EPYC-shRNA1: 5′-GACATGTATGATCTCCATCAT-3′ and LV-EPYC-shRNA2: 5′- CTAGAGGACATTCGATTGGAT-3′), and the negative control viral vector (sh-NC), were designed, constructed, and packaged by Shanghai Genechem Co., Ltd. The recombined lentiviral vector plasmid or the negative control lentiviral vector plasmid and pHelper 1.0 plasmid, the pHelper 2.0 plasmid (Shanghai Genechem Co., Ltd.) were co-transfected into 293 T cells via Hitrans G enhanced infection solution (Shanghai Genechem Co., Ltd.) at 37 °C in 5% CO2 for 6 h. The high-glucose DMEM supplemented with 10% FBS medium was refreshed. The culture supernatants were collected at 48 h following transfection. Following centrifugation at 4,000 g for 10 min at 4 °C to remove cell debris, the supernatant was filtered through 0.45 μm polyethersulfone low protein-binding filters. The concentrated viral supernatant was aliquoted and kept at − 80 °C prior to use. The lentivirus was diluted with serum-free high-glucose DMEM medium, followed by the addition of diluted lentiviral particles and polybrene (final concentration is 8 μg/ml) to PANC-1 and CFPAC-1 cells at a multiplicity of infection (MOI) of 80. Following an 8–12 h at 37 °C in 5% CO2 incubation, the medium was refreshed. Subsequently, the stable sh-EPYC cell lines of PANC-1 and CFPAC-1 were screened by purinomycin.

### Quantitative real time PCR (qPCR)

The total RNA of PCs was extracted with TRIzol reagent (Takara, 9109). All the operations were performed on ice. In a nutshell, RNA obtained above was reversely transcribed into cDNA using Prime Script™ RT Reagent Kit with gDNA Eraser (Takara, RR047A), and RT-qPCR was performed with SYBR Premix Ex Taq™ II (Takara, RR820A) on the ABI PRISM 7500 real-time PCR System according to the manufacturer's instructions. The thermocycling conditions for qPCR were as follows: 95 ˚C for 20 s, followed by 40 cycles of 95 ˚C for 5 s, 60 ˚C for 30 s and 72 ˚C for 30 s. The relative fold changes in the transcript levels were calculated using the 2^−ΔΔCT^ method. The primers used in this study were as follows respectively. GAPDH-Forward: 5′AGCCACATCGCTCAGACAC3′; GAPDH-Reverse: 5′GCCCAATACGACCAAATCC3′; EPYC-Forward: 5′ATTAGCAGGACTTGTTCT3′; EPYC-Reverse: 5′TTCTAAGGTGGCATCATA3′.

### Western blotting (WB)

All the protein samples were obtained from the centrifugation of RIPA lysis buffer (Beyotime, P0013C) containing cells. The samples were then centrifuged at 12,000×*g*, 4 °C for 30 min and supernatants were collected and stored at − 80 °C until use. After quantifying the protein concentration with a Bicinchoninic Acid protein assay kit (BCA, Thermo Fisher), we used 10% SDS-PAGE for electrophoresis to separate 20 µg proteins per sample followed by transferring the whole proteins onto nitrocellulose (NC) membranes (Beyotime, FFN63) at a constant voltage of 80 V for 2 h. Then we applied 5% skim milk to block NC membranes for 1 h at room temperature followed by incubating them with primary antibodies at 4 °C overnight. The antibodies used in this work were as follows: EPYC (Thermo Fisher, PA5-58,297, 1: 2000); PCNA (CST, 13110 T, 1:1500); E-cadherin (CST, 3195S, 1:2500); Vimentin (CST, 5741S, 1:2000); p-AKT (CST, 4060S, 1:1000); AKT (CST, 4685S, 1:3000); GAPDH (Proteintech, 10494-1-AP, 1:2000). Next day, we used a Bio–Rad chemiluminescence imaging system and a Western Bright ECL kit (Advansta, USA) to detect specific bands after NC membranes were incubated with HPR-conjugated secondary antibodies (Abcam, ab6721, 1:3000) for 1 h at room temperature. Protein bands were visualized using chemiluminescence reagent on a gel imaging system (Thermo Fisher Scientific, Inc.). The band densities were analyzed using ImageJ software (version 4.0; National Institutes of Health). The gray value of the target protein was normalized to the gray value of the internal reference protein, GAPDH, and the data are presented as the relative content of the target protein in a sample.

### Colony formation assay and cell live-dead staining assay

The colony formation assay was performed according to the protocol previously described^22^. In short, 1 × 10^3^ cells were seeded in 6-well plates and then cultured for about two weeks under different treatment conditions, and the number of colonies was finally counted. The cell live-dead staining assay was performed according to instructions of the manufacturer (YEASEN, 40747ES76). Briefly, PCs transfected with relevant plasmids were digested by trypsin and centrifuged at 500 g for 3 min. The reaction buffer (Assay Buffer) was then used to wash PCs and to make the cell suspension. Subsequently, 200 µL of cell suspension and 100 µL of staining solution were fully mixed and incubated for 15 min at room temperature. Finally, a fluorescence microscope was applied to observe cells and take pictures.

### Transwell and EdU assay

The transwell assay was performed according to the work published before^23^. In a word, PCs were seeded and cultured in the top chamber with serum-free medium (300 µL), then the complete medium (500 µL) containing 10% FBS was added into the lower chamber in a 24-well plate. After 24 ~ 48 h, we fixed PCs with 500 µL paraformaldehyde for 30 min followed by staining them with 500 µL crystal violet dye for 15 min. Finally, five random fields were selected to be photographed after the whole chambers were completely dried at room temperature. As for the EdU assay (Beyotime, C0071), all the procedures were performed according to the manufacturer’s instructions as described previously^24^.

### Subcutaneous tumor xenograft model in nude mice

The BALB/c nude mice (18~20 g, 6 weeks) were purchased from Institute of Zoology, Chinese Academy of Medical Sciences (Beijing, China). All the animal experimental procedures were approved by the Animal Care and Use Committee of Tianjin union medical center (NO. GENINK-20230007). The mice were housed under standard laboratory conditions (light cycle, 12 h dark/12 h light; temperature, 22 ± 2 °C; humidity, 55 ± 2.5%), and were provided with ad libitum access to food and water. The 1 × 10^6^ PCs suspended in 100 µL of phosphate-buffered saline (PBS) were injected into the axilla of nude mice. After 4 weeks, tumor tissues were harvested. Each tumor tissue was weighed and recorded. The euthanasia of animals progressed to cervical dislocation following an anesthetic injection (100 mg/kg ketamine and 10 mg/kg xylazine).

### Statistical analysis

The data were presented as the mean ± standard deviation, and all the assays were repeated at least 3 times independently. Statistical analyses to determine the significance between groups were performed using one-way analysis of variance (for comparisons of more than two groups) followed by Tukey's multiple comparisons test or an unpaired Student's t-test (for comparisons between two groups). All the analyses were performed using SPSS 26 for Windows (SPSS, Inc., Chicago, IL, USA) and GraphPad Prism (Version 6.0) for Windows (GraphPad Software, Inc., San Diego, CA, USA). *P* < 0.05 was considered to indicate statistical significance (^*^p < 0.05; ^**^p < 0.01; ^***^p < 0.001; n.s., not significant).

### Consent to participate and ethics statement

All the animal experiments in this work were approved by the Animal Care and Use Committee of Tianjin union medical center of Nankai University. All methods were performed following the relevant guidelines and regulations. The Ethics Number: GENINK-20230007.

## Results

### The preliminary analysis of raw data, visualization of DEGs and functional enrichment analysis of target genes

**Figure 1:**
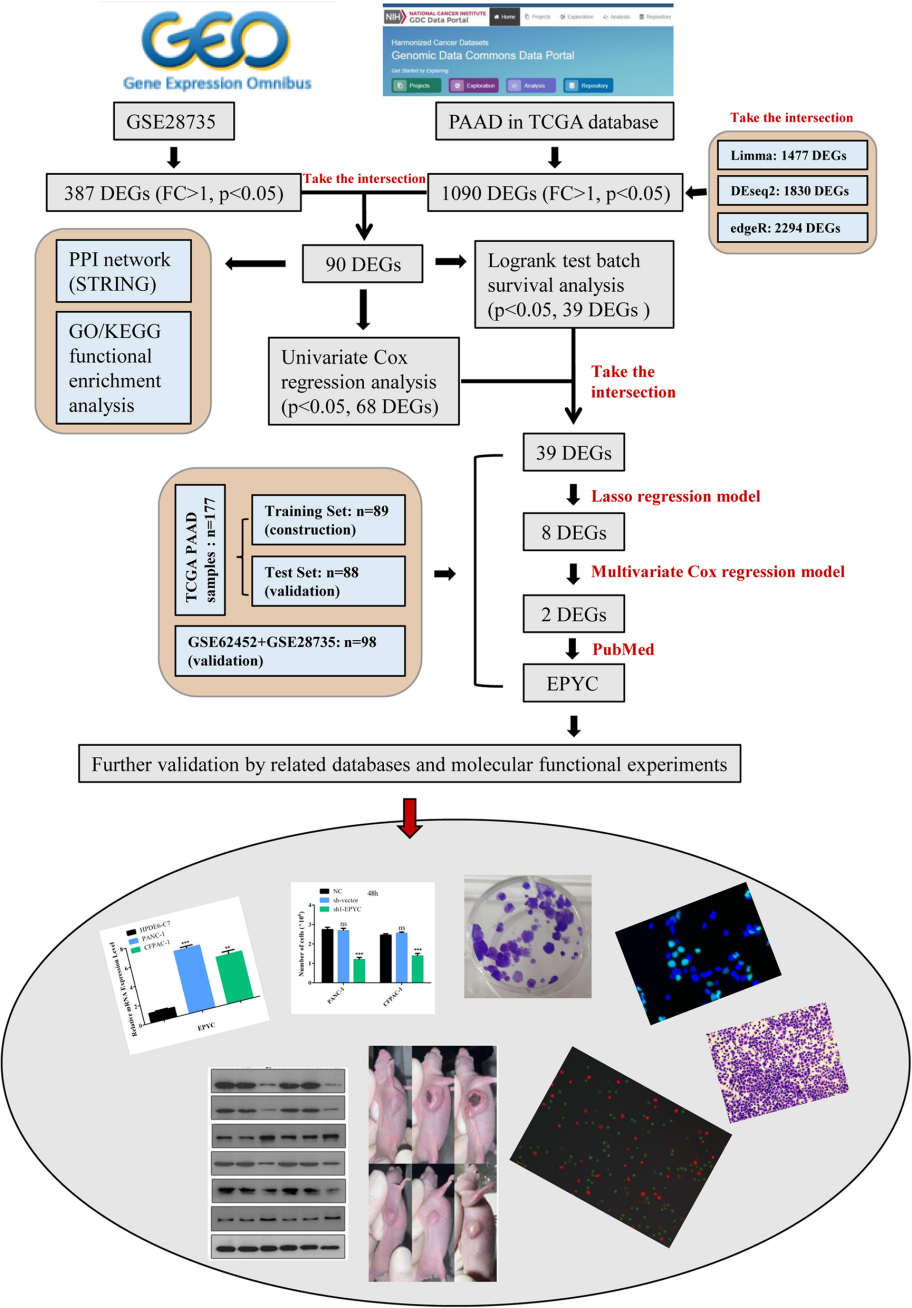
The workflow of this study. Detailed operation process from data download to screening of candidate genes and final validation.

**Figure 2:**
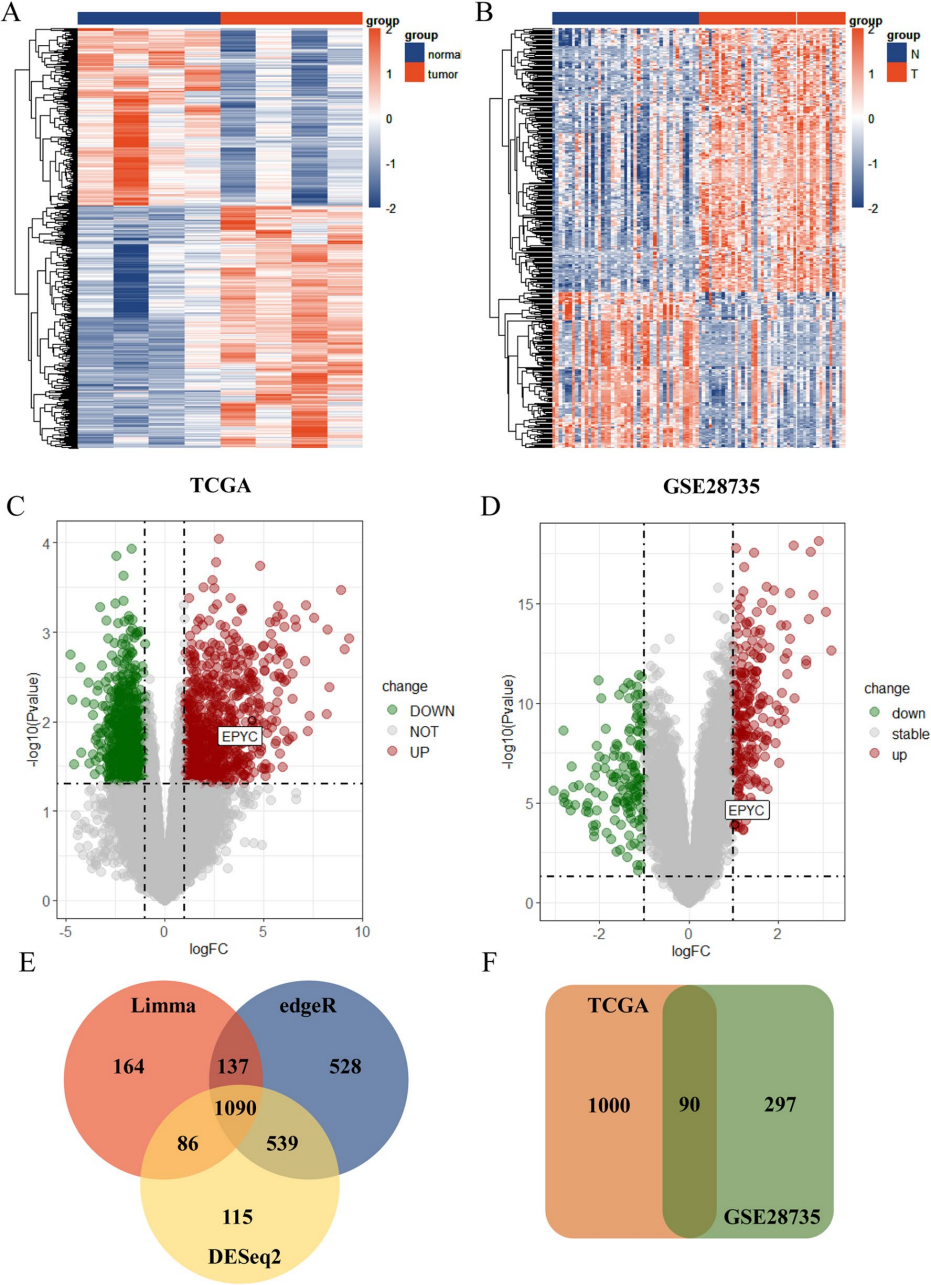
The visualization of DEGs. (**A**, **B**) The heatmap for TCGA samples and GSE28735 samples. (**C**, **D**) The volcano map for TCGA samples and GSE28735 samples. (**E**) The venn diagram for the intersection of DEGs calculated by three R packages in TCGA samples. (**F**) The venn diagram for the intersection of DEGs from TCGA samples and GSE28735 samples.

**Figure 3:**
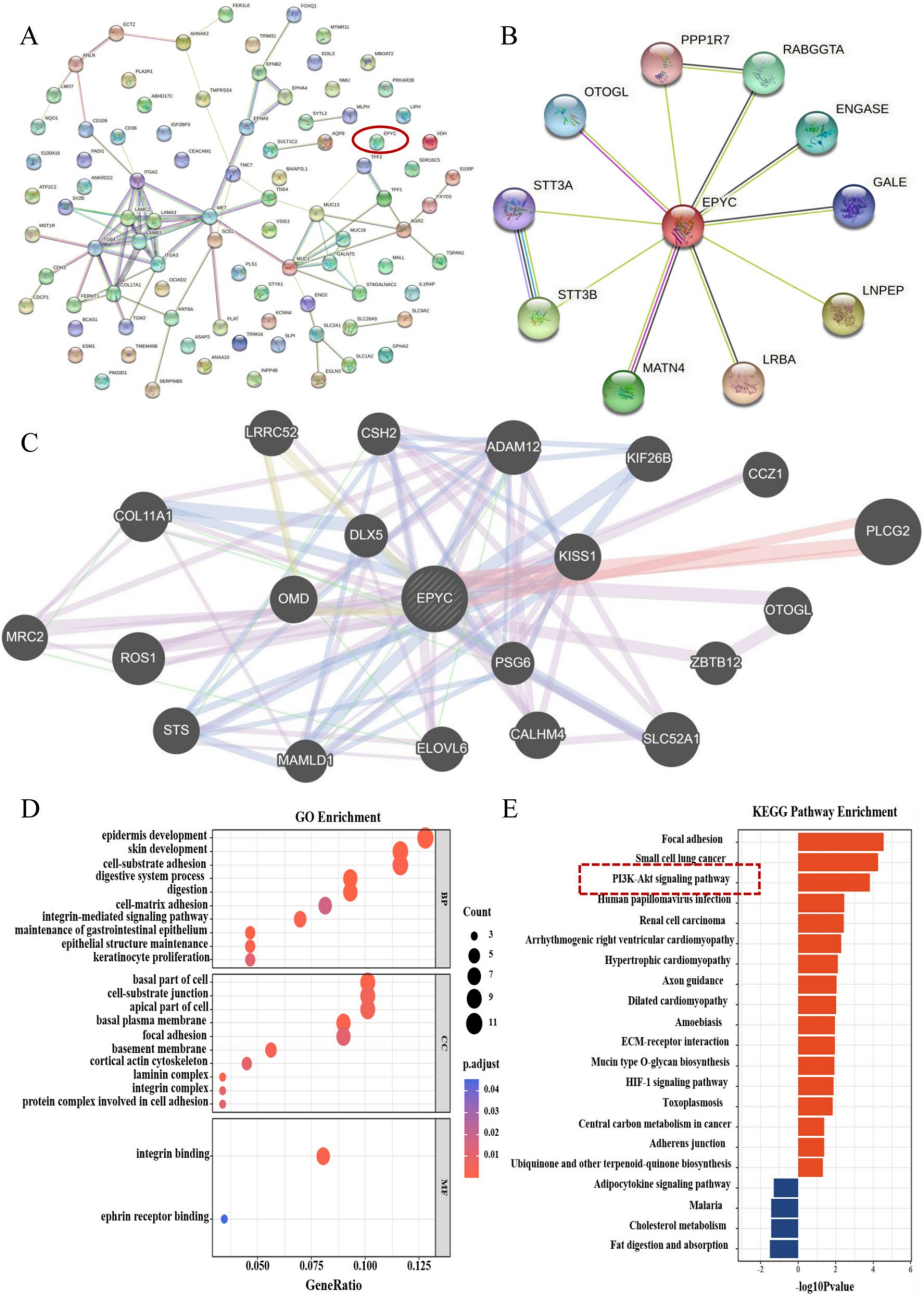
The interaction protein profile and functional enrichment analysis of EPYC. (**A**) The PPI network of all the candidate genes from STRING online website. (**B**) The PPI network of EPYC from STRING online website. (**C**) The PPI network of EPYC from GeneMANIA online website. (**D**, **E**) The GO and KEGG enrichment analysis for the whole candidate genes.

In order to discover more instructive biological signatures for the clinical treatment of PC, we downloaded relevant sample expression matrices from TCGA and GEO online databases followed by performing a series of analysis and validation. The overall operational analysis flow was shown in Fig. 1. Then we handled a preliminary processing of the downloaded data and filtered out genes with zero expression in all samples. The results of differential expression analysis revealed 1090 DEGs in TCGA samples (Fig. 2A) and 387 DEGs in GSE28735 samples (Fig. 2B). Furthermore, we also labeled the final candidate gene EPYC separately in volcano maps, and the results showed that EPYC was up-regulated in tumor tissues compared with that in normal tissues (Fig. 2C,D). To further narrow down the candidate genes, we took the intersection of differential analysis results from TCGA samples processed respectively by three different R packages to finally focus on 1090 DEGs (Fig. 2E). Then the above results were intersected with the results of differential analysis on GEO samples, and finally 90 candidate DEGs were obtained for subsequent analysis (Fig. 2F). For purpose of understanding how these 90 DEGs are related to each other, we constructed a PPI network in String online website, and the results described that most of these genes were not linked to each other including EPYC (Fig. 3A). Therefore, we singled out the PPI network of EPYC using String and GeneMANIA databases to indirectly understand the role of EPYC (Fig. 3B,C). To further clarify which biological processes these 90 DEGs are involved to modulate, we performed the functional enrichment analysis (Fig. 3D,E). Interestingly, we noticed that the PI3K-AKT classical molecular signaling pathway was significantly enriched in elevated DEGs at this step. In addition, we used the GEPIA database to analyze the expression levels of EPYC and the related protein levels of PI3K-AKT pathway in PC including AKT1, AKT3, PIK3CA, PIK3R1 and PIK3R2. The results showed that the level of EPYC was positively correlated with the PI3K signaling pathway (Fig. S1A–E).

### The establishment and validation of a lasso regression model

**Figure 4:**
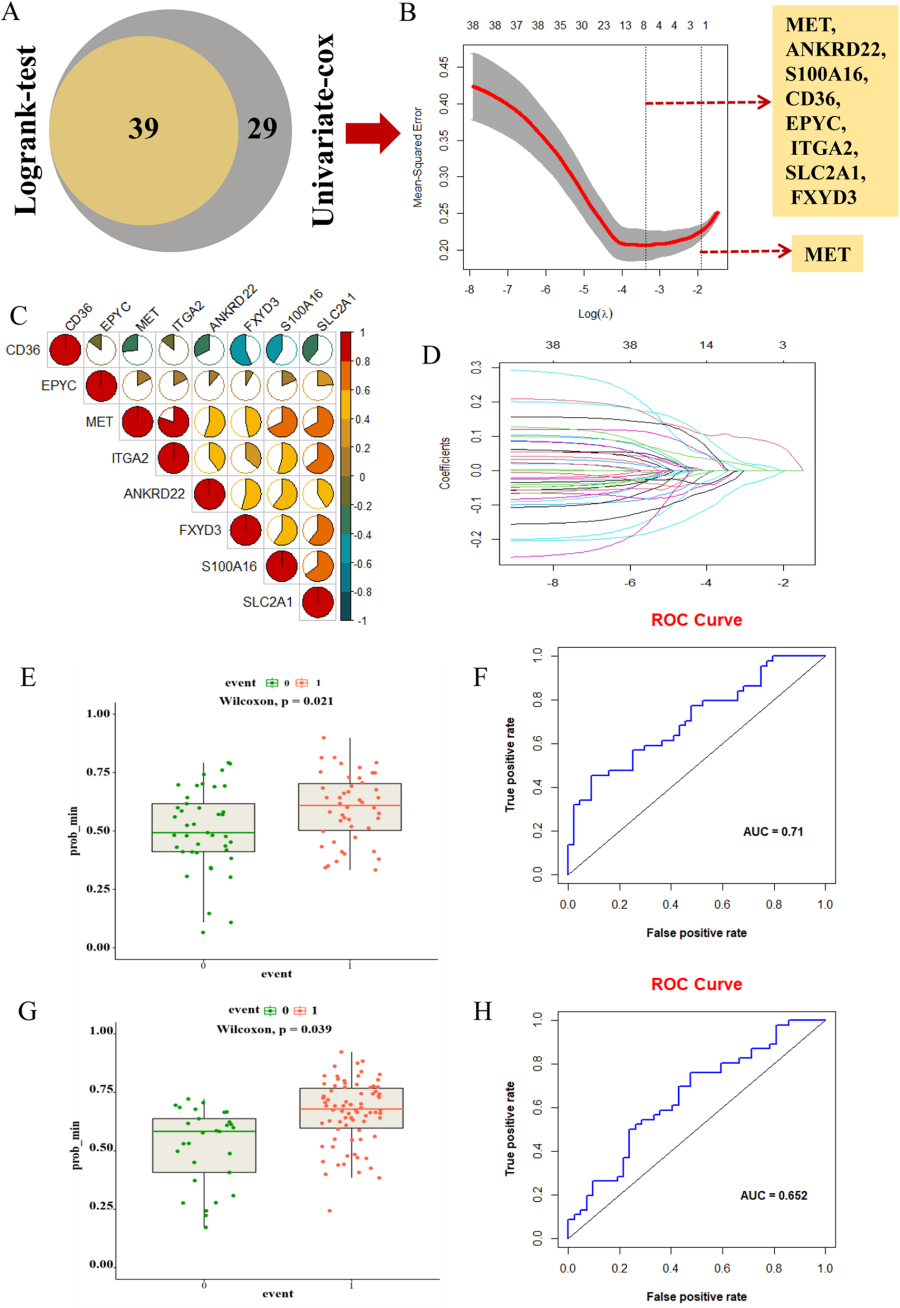
The construction and verification of a lasso regression model. (**A**) The Venn Diagram for the intersection of DEGs obtained respectively by log-rank test analysis and univariate cox regression analysis. (**B**) The lasso regression model established by the train group data for calculating 1000 lambda values to select the best performing value. The vertical dotted lines were drawn at the optimal values with the min and 1se criteria. The criteria of min corresponded to 8 genes, and the criteria of 1se corresponded to only gene MET. (**C**) The correlation heatmap for 8 candidate genes screened out with the criteria of min. (**D**) The coefficient graph of the lasso regression model. (**E**) The validation for the lasso model by test group data. Each point represents the true survival status of patients (0, alive; 1, dead). The ordinate represents the predicted survival status. (**F**) The ROC curve for the test group data. (**G**) The validation for the lasso model by GEO data (GSE62452 and GSE28735). (**H**) The ROC curve for the GEO data.

**Figure 5:**
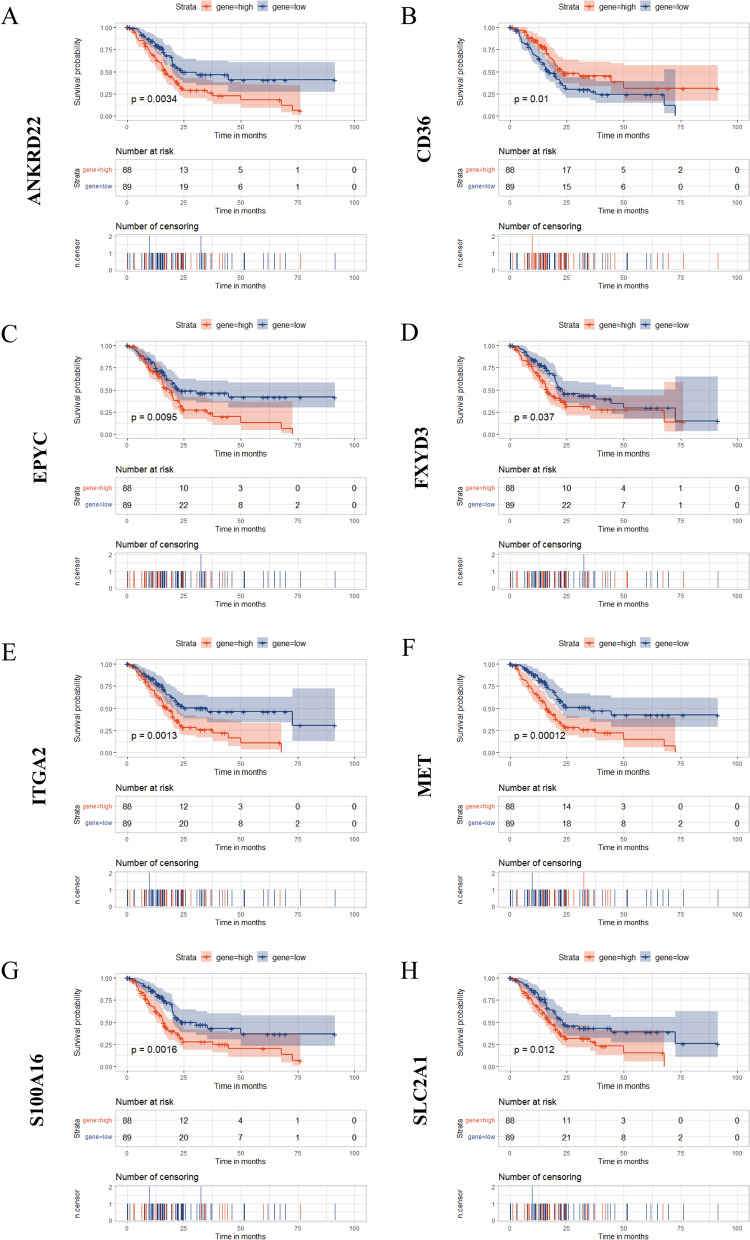
The survival curves of candidate 8 genes in TCGA samples. (**A**) The survival curve of ANKRD22. (**B**) The survival curve of CD36. (**C**) The survival curve of EPYC. (**D**) The survival curve of FXYD3. (**E**) The survival curve of ITGA2. (**F**) The survival curve of MET. (**G**) The survival curve of S100A16. (**H**) The survival curve of SLC2A1.

To further prick off clinically instructive genes, we operated further bulk survival analysis on the 90 DEGs obtained previously. As shown in Fig. 4A, the result of a log rank-test analysis showed that 68 genes (*p* < 0.05) exerted statistically significant effects on patient survival (Fig. 4A). In addition, we also performed the univariate cox regression analysis on these 90 DEGs and finally obtained 39 genes *(p* < 0.05), then the results of above two analyses were intersected to acquire 39 candidate genes for subsequent analysis. We then established a lasso regression model with the training set of TCGA samples, and the results exhibited two extreme λ values corresponding to 8 genes (MET, ANKRD22, S100A16, CD36, EPYC, ITGA2, SLC2A1, FXYD3) and 1 gene (MET) respectively (Fig. 4B,D). In the meantime, we also made a correlation analysis on the expression of these 8 candidate genes, and the results manifested that the correlation between them was all relatively low (Fig. 4C). To verify the feasibility of the previous lasso regression model, we substituted the data from the test set of TCGA samples into the model, and the results illustrated that the true survival status of patients was generally consistent with the predicted value (Fig. 4E). What’s more, the area under the ROC curve also reached 0.71 (Fig. 4F), which we believed was applicable. Afterwards, we also validated the model with data from GEO samples, and the results were also as expected (Fig. 4G,H). For the sake of further identification of specific targets, we selected these 8 genes with significant survival significance as candidate genes for building follow-up models. High expression of EPYC, ANKRD22, ARNTL2, DSG3, KRT7, PRSS3 and MET predict poor prognosis of pancreatic cancer patients. However, high expression of CD36 predicts a good prognosis for pancreatic cancer patients (Fig. 5A,H). In summary, we constructed and validated a lasso regression model to finally screen out 8 genes as new candidate biological targets for PC.

### The establishment and validation of a multivariate cox proportional hazards regression model

**Figure 6:**
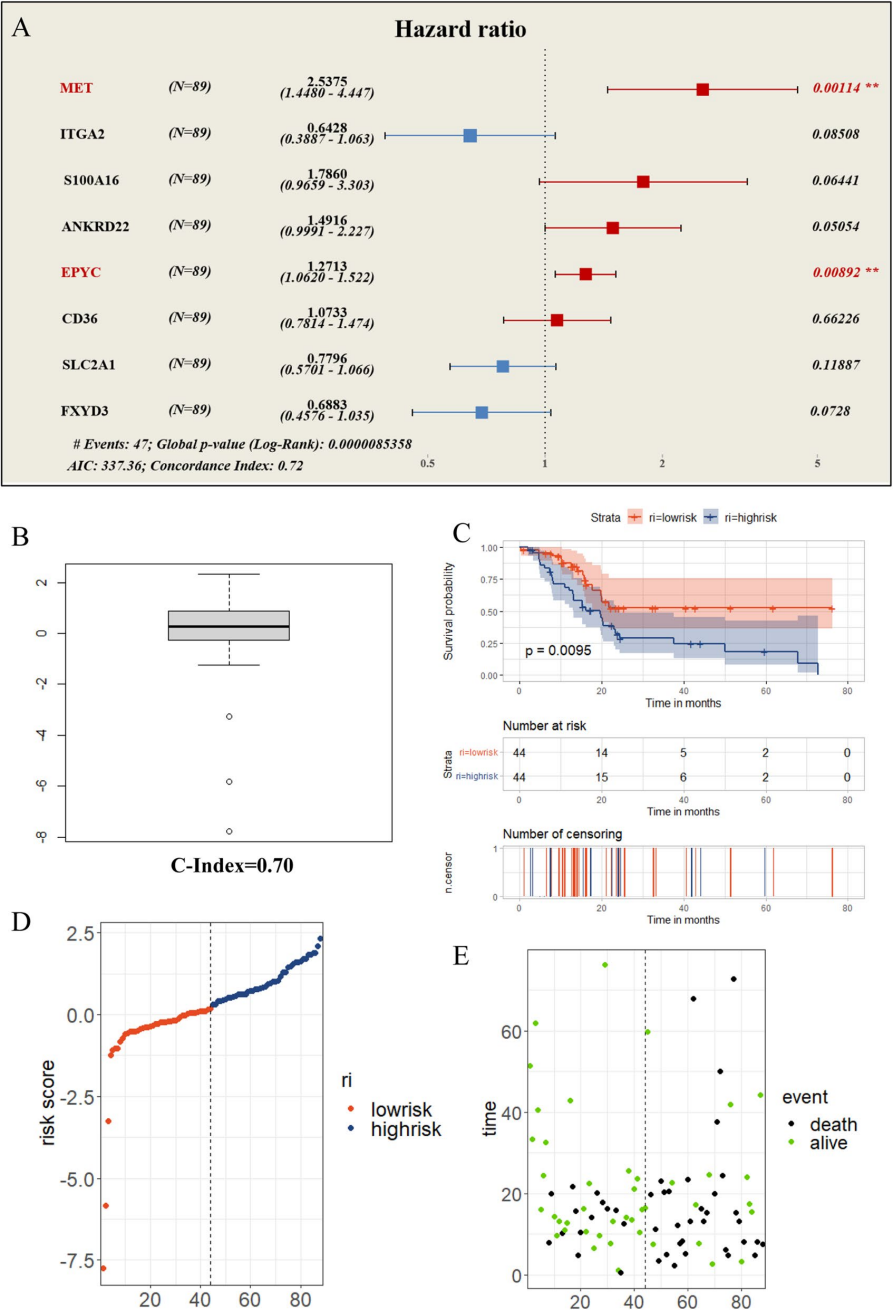
The construction and validation of a multivariate cox regression model. (**A**) The risk forest diagram for the visualization of the multivariate cox regression model constructed by train group data. (**B**) The c-index prediction boxplot diagram of test group data. (**C**) The high-low risk survival curve of the predictive model generated using test group data. (**D**) The distribution of risk scores in test group. Each point represents a patient sample. (**E**) The true survival status of each patient sample corresponding to panel D one-to-one.

**Figure 7:**
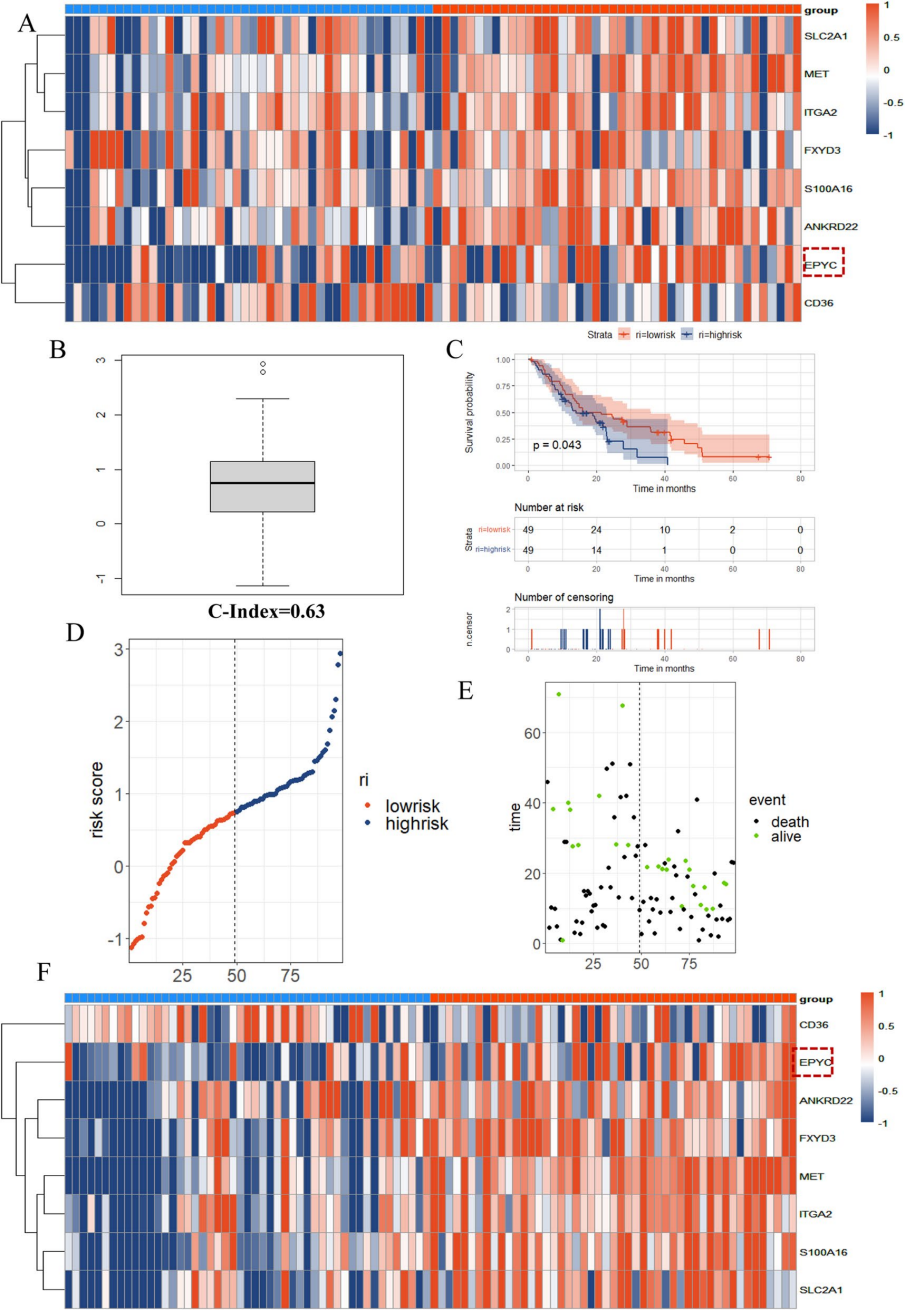
Validation of the multivariate cox regression model. (**A**) The heatmap of 8 candidate genes for each patient in test group. (**B**) The c-index prediction boxplot diagram of GEO data. (**C**) The high-low risk survival curve of the predictive model generated using GEO data. (**D**) The distribution of risk scores in GEO data group. (**E**) The true survival status of each patient sample corresponding to panel D one-to-one. (**F**) The heatmap of 8 candidate genes for each patient in GEO data group.

To further clarify whether the above 8 candidate genes could be independent risk factors for PC, we constructed and visualized a multivariate cox regression model with the training set of TCGA samples. As shown in Fig. 6A, we observed that 5 genes (MET, ANKRD22, S100A16, CD36, EPYC) were deemed as risk factors and 3 genes (ITGA2, SLC2A1, FXYD3) were regarded as protective factors in this model (Fig. 6A). However, the risks of MET and EPYC were considered to be statistically significant. Therefore, we could tentatively identify MET and EPYC as independent risk factors for PC. After reviewing relevant literatures, we found that the roles of MET in PC had been reported by some research teams^25–28^, so we set our final target on EPYC. Next, we also verified the availability of this model with the test set of TCGA samples. On the basis that the c-index value of training set was only 0.72, the c-index value of test set could also reach 0.70 (Fig. 6B), which was sufficient to declare that this multivariate cox regression model possessed certain reference significance. Simultaneously, we also divided the test set data into two groups (high-risk and low-risk) according to the predicted values followed by performing the survival analysis, and the results presented a statistically significant difference in survival status between two groups (Fig. 6C). In addition, we also put 3 indicators (risk value, survival status, gene expression level) of each patient in one-to-one correspondence, and the results revealed that patients in the high-risk group had the higher true mortality rate (Fig. 6D,E) as well as the higher expression level of EPYC (Fig. 7A) compared with those in the low-risk group. Similarly, we performed the same validation of the GEO data and received the analogous trend of results, indicating the good usability of the model again (Fig. 7B–F). All seven genes except EPYC have been extensively studied in pancreatic cancer. Here, we constructed and validated a practicable multivariate cox regression model to screened out EPYC as an independent risk factor for PC.

### *EPYC promoted the proliferation and survival of PCs *in vitro

**Figure 8:**
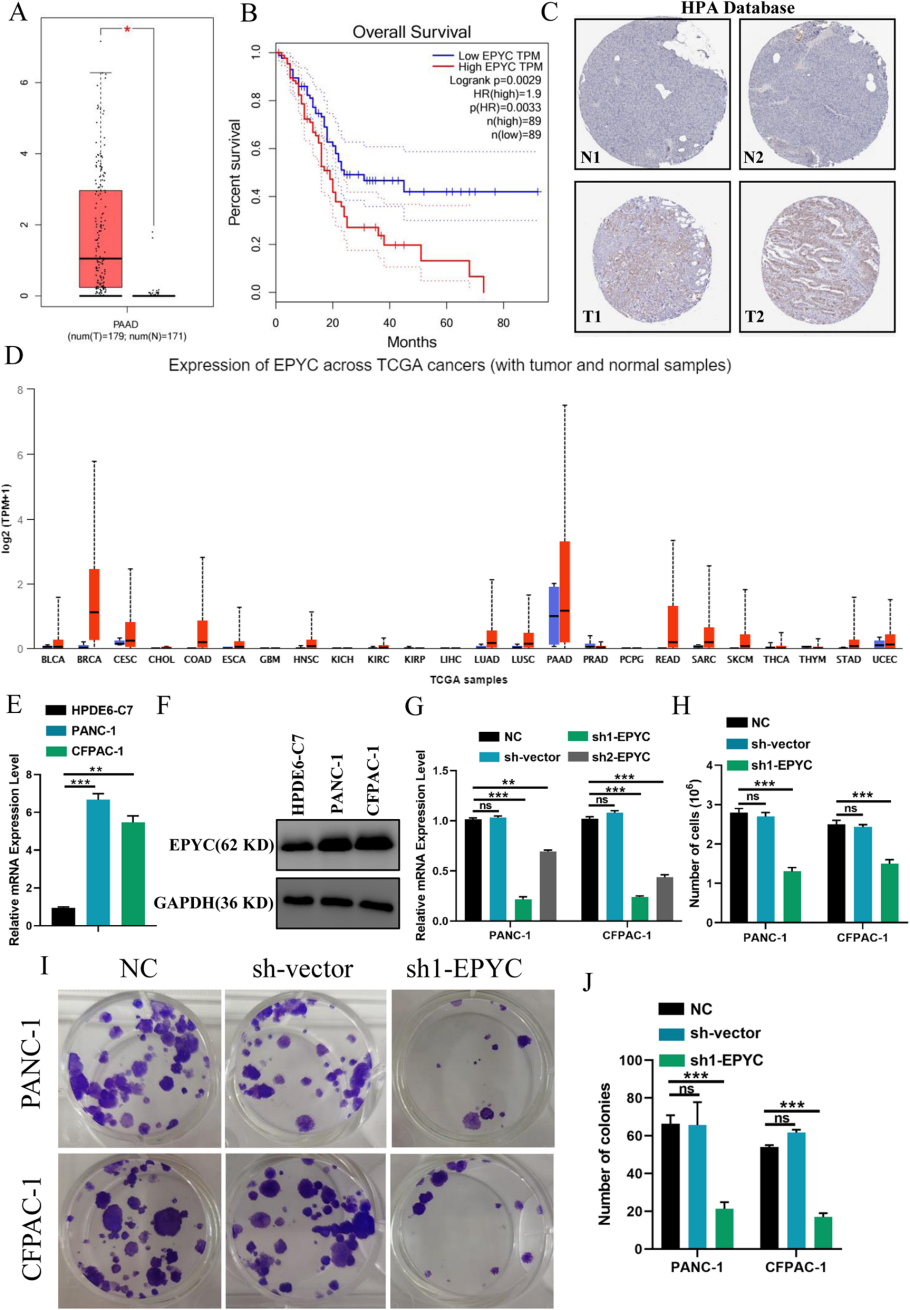
EPYC was highly expressed in pancreatic cancer. (**A**) The expression histogram of EPYC in normal and tumor tissues from GEPIA database. (**B**) The survival curve of EPYC from GEPIA database. (**C**) Representative images of immumohistochemical staining of EPYC in normal and tumor tissues from HPA database. (**D**) Pan-cancer analysis of EPYC from ULCAN database. (**E**) The mRNA expression level of EPYC by qPCR assay. (**F**) The protein expression level of EPYC by western blot assay. (**G**) The effectiveness of the plasmid is shown. (**H**) Cell counts in PCs transfected with or without related plasmids for 48 h. (**I**) Representative images of the proliferation ability of PCs by colony formation assay. (**J**) Quantification of the data shown in H. Results were presented as mean ± s.d. Student’s t-test was used to analyze the data. (^*^p < 0.05; ^**^p < 0.01; ^***^p < 0.001; n.s., not significant).

**Figure 9:**
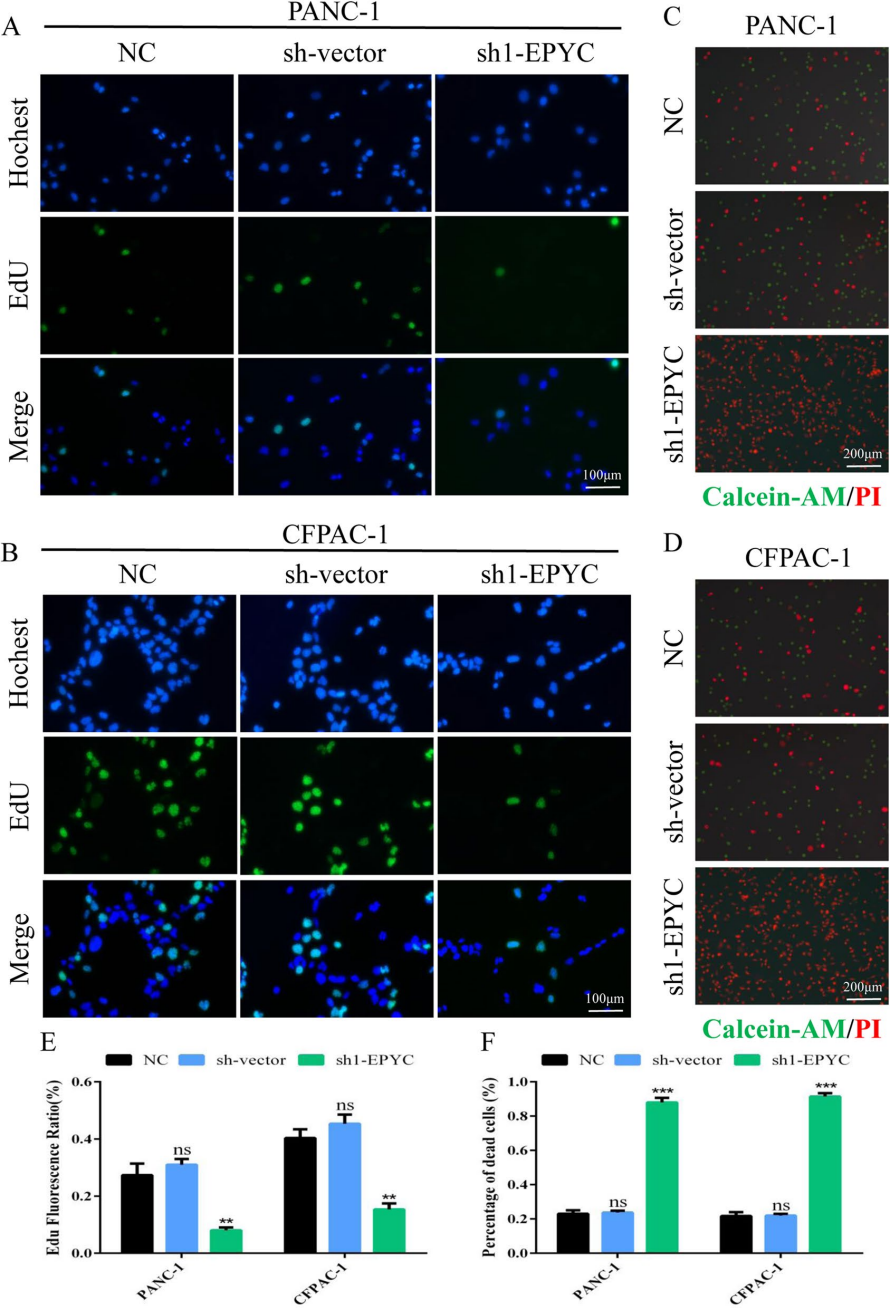
EPYC promoted the proliferation and survival of PCs in vitro. (**A**, **B**) EdU incorporation assay was performed to detect the proliferation of PANC-1 and CFPAC-1. (**C**, **D**) Live-dead staining assay was performed to detect the survival status of PANC-1 and CFPAC-1. (**E**) Quantification of the data shown in A and B. (**F**) Quantification of the data shown in C and D. Results were presented as mean ± s.d. Student’s t-test was used to analyze the data. (^*^p < 0.05; ^**^p < 0.01; ^***^p < 0.001; n.s., not significant).

In order to explore the potential mechanism of EPYC in PC, we next performed a series of molecular functional assays. To further confirm the role of EPYC in PC, we queried EPYC data in relevant online databases (GEPIA, HPA, ULCAN), and the results showed that EPYC functioned as an oncogene to be highly expressed in tumor tissues and had a significant impact on the survival prognosis (Fig. 8A–D). We next examined the expression level of EPYC at the RNA level in two PCs, and the results demonstrated that EPYC was highly expressed in PCs compared to that in HPDE6-C7 cells (Fig. 8E). In addition, the protein levels of EPYC were also up-regulated in PC cell lines compared with HPDE6-C7 (Fig. 8F). Then we designed two EPYC knockdown sequences and verified their efficiency in PCs (Fig. 8G). To investigate the influence of EPYC on the proliferation of PCs, we performed CCK8 to identify the cell proliferation in PCs. The results showed that sh1-EYPC had the highest knockdown efficiency and the strongest inhibition on cell proliferation at 24 h and 48 h (Fig. S2A,B). So, we selected the sh1-EPYC for subsequent work. We counted the cells after the planking for 48 h, and the results showed that knocking down EPYC could significantly reduce the number of PCs (Fig. 8H). Meanwhile, the results of colony formation assays showed that PCs with decreased EPYC had fewer colonies (Fig. 8I,J). In addition, the results of EdU assays showed that down-regulated EPYC inhibited the proliferation in PANC-1 and CFPAC-1 cells (Fig. 9A,B,E). By combining all the above results, we confirmed that EPYC could promote the proliferation of PCs in vitro.

### *EPYC promoted the migration of PCs *in vitro* and the proliferation of PCs *in vivo

**Figure 10:**
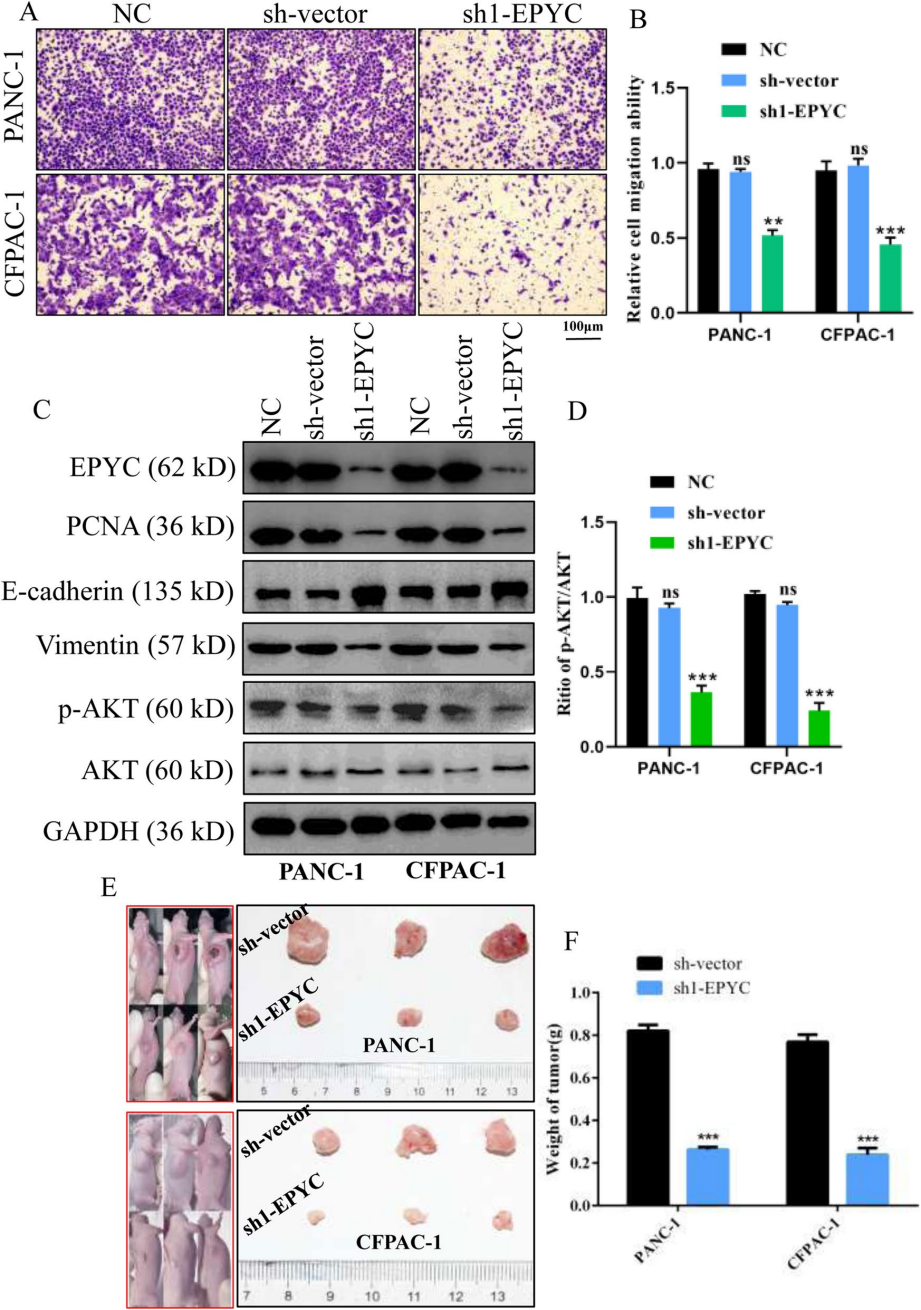
EPYC promoted the migration of PCs in vitro and the proliferation of PCs in vivo. (**A**) Representative images of the migration ability of PCs by Transwell assay. (**B**) Quantification of the data shown in A. (**C**, **D**) Western blot was performed to detect designated proteins in PCs transfected with specific plasmids. (**E**) Schematic diagram of subcutaneous tumor formation. Tumors derived from PCs were displayed. (**F**) Quantification of tumor weight from E. Results were presented as mean ± s.d. Student’s t-test was used to analyze the data. (^*^p < 0.05; ^**^p < 0.01; ^***^p < 0.001; n.s., not significant).

To further explore the function of EPYC in PC cells, we also examined its effect on the survival and migration of PCs in vitro. As shown in Fig. 9C,D and Fig. 9F, the results of the live-dead staining assay showed that lessened EPYC could promote the death of PCs (Fig. 9C,D,F). However, the results of Transwell assay demonstrated that reduced EPYC suppressed the migration of PCs (Fig. 10A,B). In addition, knockdown of EPYC could reduce the expression level of PCNA and Vimentin but elevate the expression level of E-cadherin, which further suggested that EPYC could promote the proliferation and migration of PCs. Meanwhile, we noticed that the expression level of p-AKT was also reduced along with the decrease in EPYC, indicating that EPYC exerted the function in PCs probably partly via PI3K-AKT signaling pathway (Fig. 10C,D). To further confirm our hypothesis, we performed a subcutaneous nude mice tumorigenesis assay to verify the function of EPYC regarding promoting the proliferation of PCs in vivo, and the results made clear that the EPYC knockdown group had smaller masses compared to that in control group (Fig. 10E,F). In conclusion, we substantiated the hypothesis that EPYC could promote the proliferation and migration of PCs in vitro and vivo, therefore we declared EPYC as a novel biological signature for PC in this work.

## Discussion

Pancreatic cancer is a disease with a poor prognosis in clinical, and the 5-year survival rate is still very low today^29–31^. Its disease characteristics (difficulties in early diagnosis, rapid aggressiveness, high therapy resistance, etc.) pose great challenges to clinicians in overcoming PC^32–34^. Once a patient is diagnosed with PC, the tumor is basically at an advanced stage^35^. Meanwhile, current treatments and outcomes for PC are limited, which results in a very high lethality ratio. Based on these above reasons, this work aimed to discover more instructive biological biomarkers followed by providing fresh directions for early diagnosis and later treatment of PC.

Recently, Zhang et al. reported that UTX was a potential biomarker for PDA and functions as a tumor suppressor gene, and inhibited PDA growth and metastasis. Mechanistically, GATA6 activated UTX expression by direct binding to its promoter region and activating its transcription. The UTX-GATA6 signaling axis offers a potentially novel therapeutic target for PDA treatment ^36^. Huang et al. used The Cancer Genome Atlas (TCGA) database to obtain the transcriptome and clinical data of PAAD. The optimal lncRNA was screened by Cox and the Least Absolute Shrinkage and Selection Operator (LASSO) regression mode, and for the construction of risk scoring system. They found that lncRNA scoring system (CRLss) could predict the clinical outcome and immune landscape of PAAD patients, identify the potential beneficiaries of immunotherapy, and provide a reference for precise therapeutic drug selection ^37^.

In this study, we obtained 8 candidate genes including CD36, EPYC, ANKRD22, FXYD3, MET, ITGA2, S100A16 and SLC2A1 via bioinformatics analysis, and the follow-up analysis was performed to further screen out independent risk factors. Tang et al. reported that high CD36 expression is linked to poorer survival in patients receiving postoperative AG using the tissue microarray analysis. Targeting CD36 synergistically improves the PDAC response to AG both in vitro and in vivo, including patient-derived preclinical models ^38,39^. A machine learning based-study reported that the 5-gene signature including ANKRD22, ARNTL2, DSG3, KRT7, PRSS3 performed well on both our chosen training dataset and validation dataset and provided a new way to predict the prognosis of pancreatic cancer patients ^40^. FXYD3 was expressed in human pancreatic tissues, with a significant upregulation in at least 50% of PDAC tissues, due mainly to increased expression levels within the cancer cells themselves. Downregulation of FXYD3 in pancreatic cancer cell lines altered its growth behavior both in vitro and in vivo, suggesting that FXYD3 also maybe a risk factor in PC ^41^. An acceptable survival prognosis was demonstrated by ITGA2, which was highly expressed during in vitro PDAC cell proliferation, apoptosis, and migration and ITGA2 was potential prognostic biomarkers for PDAC associated with IPMN ^42^. S100A16 was overexpressed in PDAC and promoted PDAC progression through FGF19-mediated AKT and ERK1/2 signaling, suggesting that S100A16 may be a promising therapeutic target for PDAC ^43^. Survival analysis of TCGA data and clinical data suggested that SLC2A1 can be potential biomarkers for the prognosis of PC ^44^. However, the role of EPYC in PC was not clear.

Although our research results demonstrated that some genes were not independent risk factors, we believed they also exerted vital effects on PC, yet their exact roles still deserved further researches. Similarly, the results of pan-cancer analysis suggested that EPYC was aberrantly expressed in many tumors (breast cancer, colon adenocarcinoma, lung adenocarcinoma, etc.) in addition to PC, whereas the roles of EPYC in these tumors were still obscure. We found that EPYC promoted cell viability and proliferation in vitro. In addition, we also reported that EPYC could regulate the PI3K-AKT pathway through the bioinformatic analysis and the indicated experiments. However, which upstream genes can regulate EPYC or which downstream genes of EPYC are involved in the regulatory process are still very ambiguous and need to be further explored. At the same time, we established the subcutaneous tumorigenesis model in nude mice and elucidated that EPYC could also promote the proliferation of PC in vivo, but we guessed that the evidence was still not sufficient enough. Due to the constraints of time and funding, further constructing in situ tumor transplantation models or transgenic mouse models were not be fulfilled. We hope that our team will have the ability to further explore the remaining issues in this work and further confirm the role of EPYC in PC via subsequent studies.

At this point, we have initially explored the role played by EPYC in PC in this article and provided more possibilities for the diagnosis and treatment of PC. Although here we reported that EPYC might serve as a prognostic biomarker for PC, there was still a long way to verify whether this gene could actually be used as a therapeutic target of PC in the clinic. Nonetheless, we have made an appropriate contribution to fight against PC.

## Conclusion

Based on the above results of integrated bioinformatics analysis and molecular functional assays, we fortunately draw a conclusion that EPYC could promote the malignant progression of PC and function as a novel prognostic signature.

### Supplementary Information

Supplementary Information.

## Data Availability

The data sets analyzed during the current study are available from the corresponding author on reasonable request. This study is reported in accordance with the ARRIVE guidelines.
