# Supplementary material for: EPYC functions as a novel prognostic biomarker for pancreatic cancer
Source: Sci Rep. 2024 Jan 6;14:719. doi: 10.1038/s41598-024-51478-w (PMC10771449; doi:10.1038/s41598-024-51478-w)

**EPYC functions as a novel prognostic biomarker for** **pancreatic cancer**

Zhen Yang^1,2#*^, Honglin Li^3#^, Jie Hao^4#^, Hanwei Mei^2,5^, Minghan Qiu^2,5^, Huaqing Wang^2,5*^and Ming Gao^4*^.

**Figure S1**


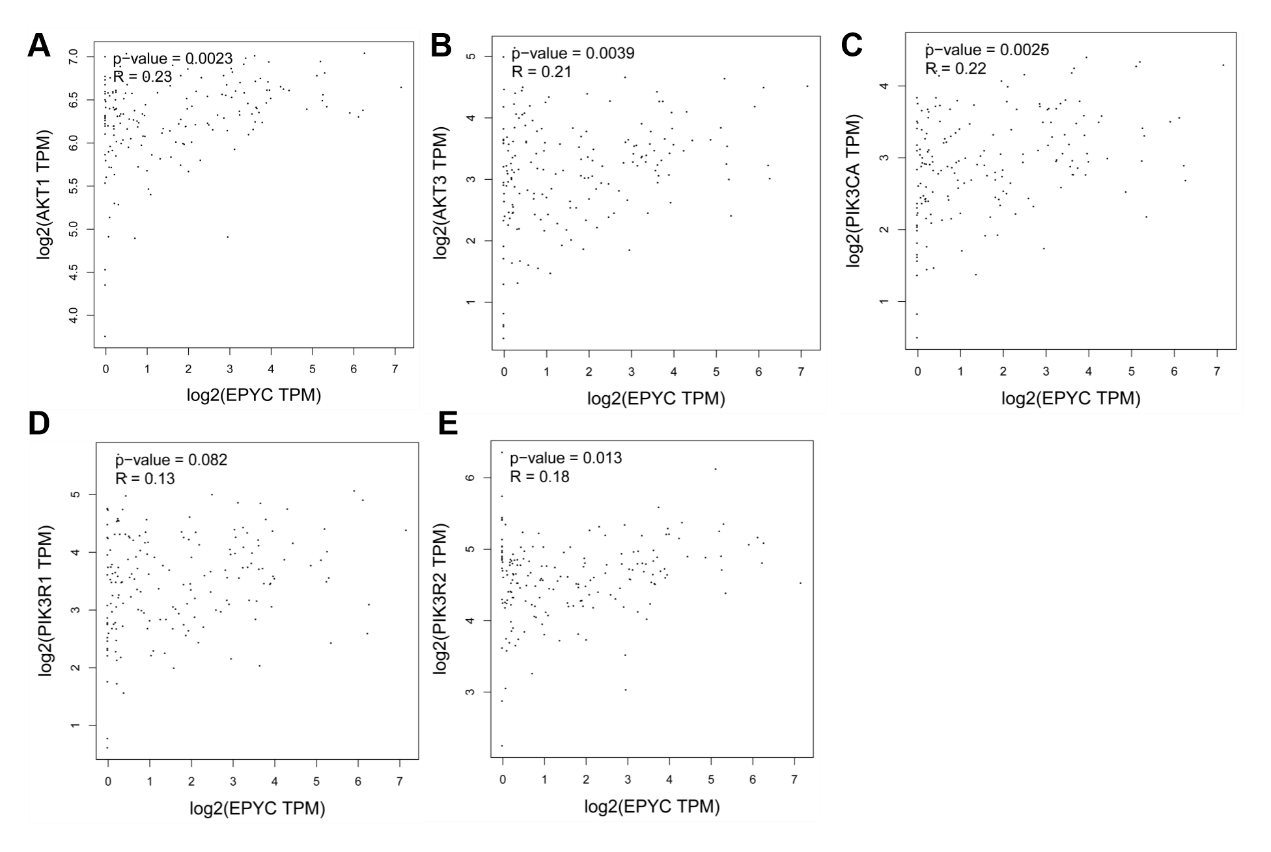


Figure S1 The expression level of EPYC was positively correlated with the PI3K-AKT pathway including AKT1 (A), AKT3 (B), PIK3CA (C), PIK3R1 (D) and PIK3R2 (E).

**Figure S2**


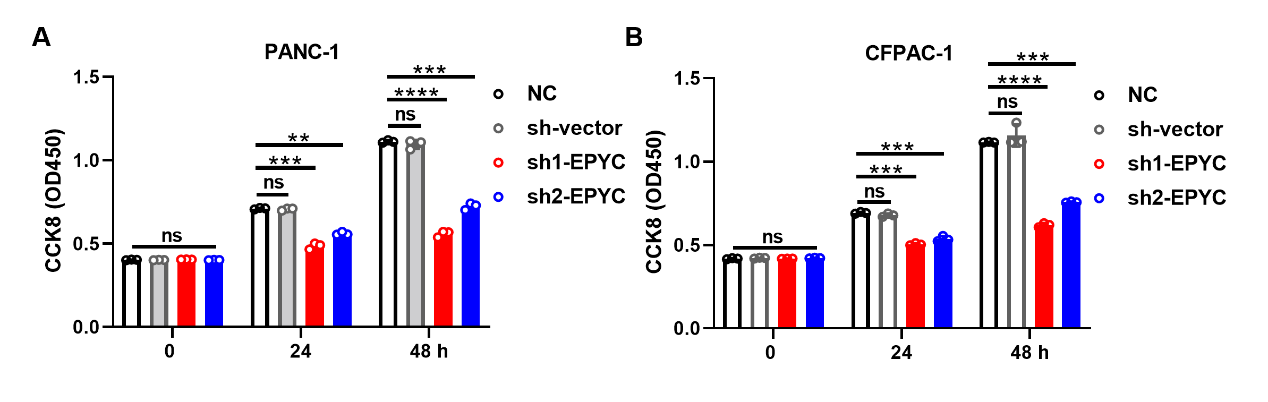


Figure S2 CCK8 assay showed the cell proliferation. (A) CCK8 showed the role of EPYC on cell proliferation in PANC-1 cells. (B) CCK8 showed the role of EPYC on cell proliferation in CFPAC-1 cells.

**Western Blot raw data**


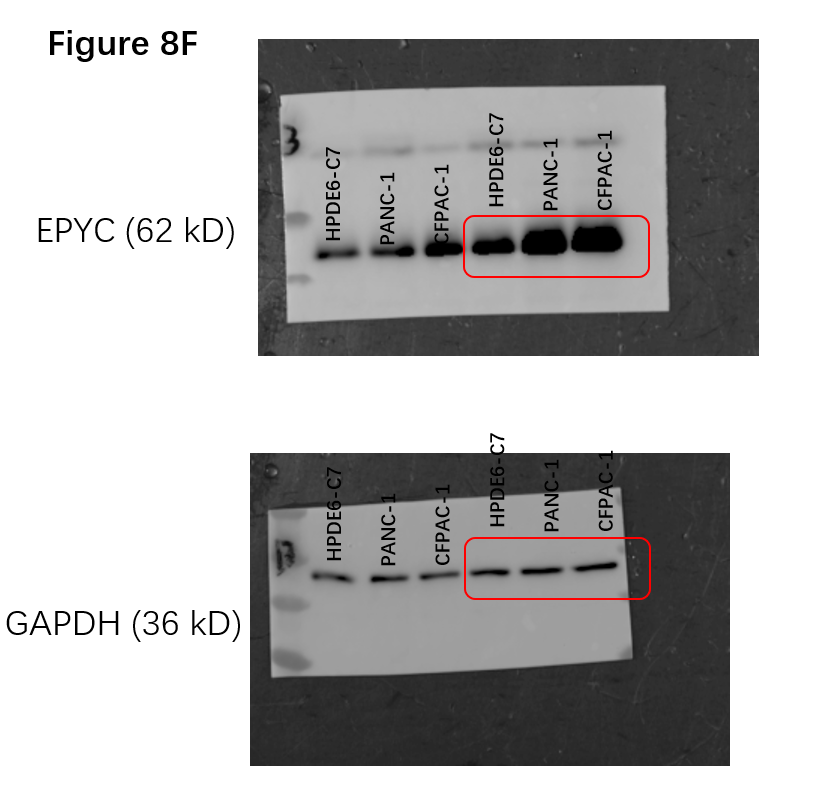


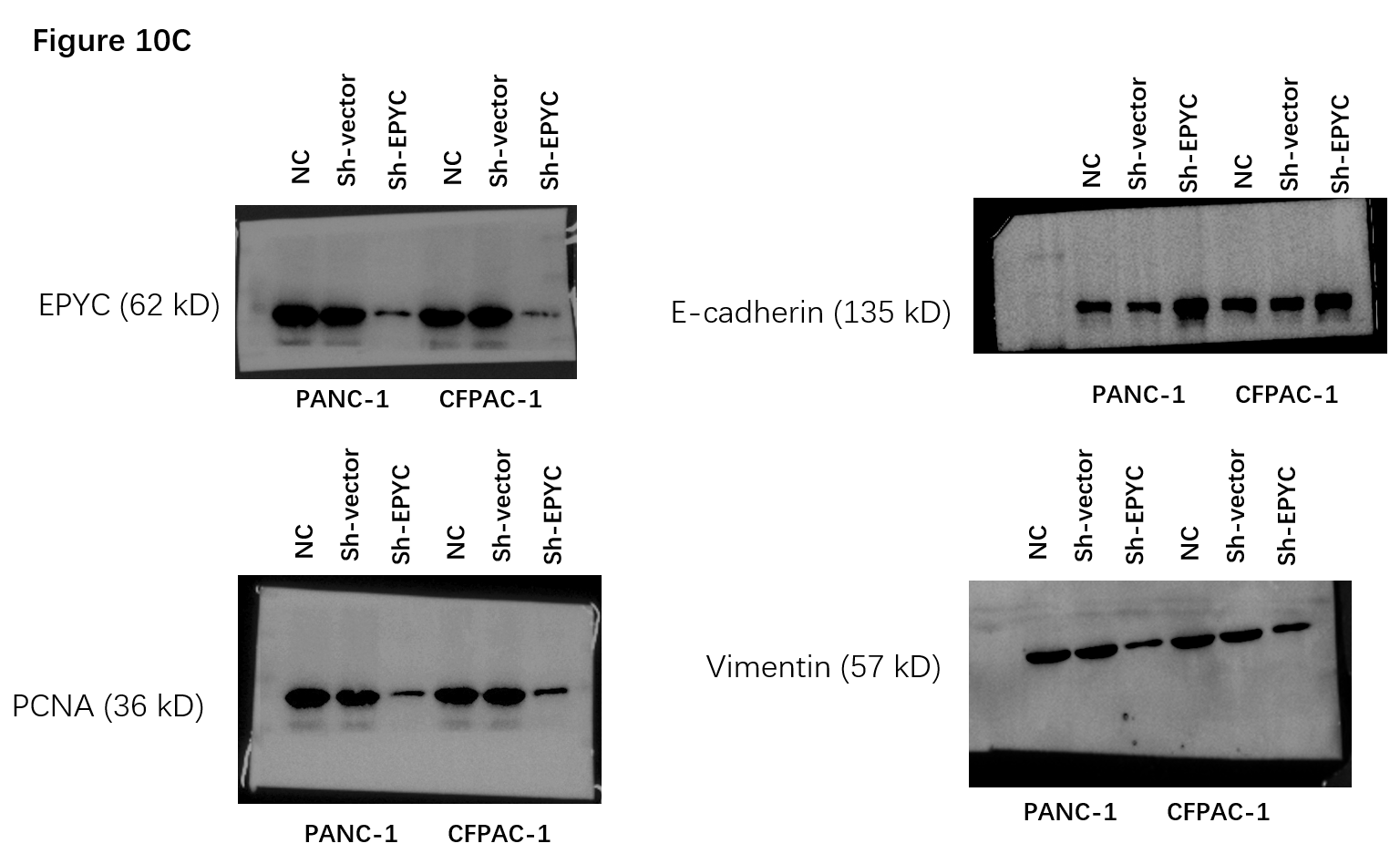


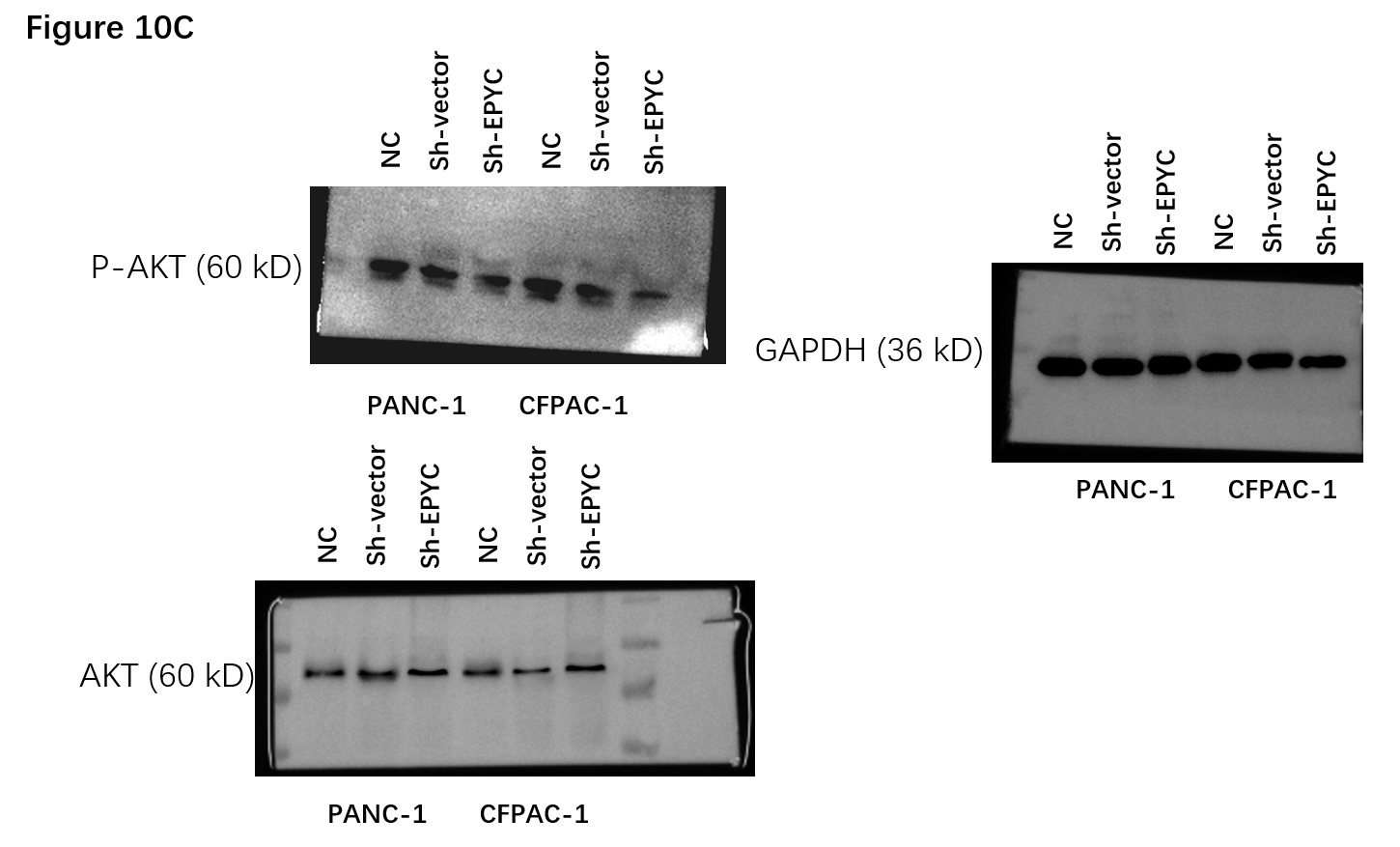

Supplement: Supplementary file 1 — Supplementary Information. [file 41598_2024_51478_MOESM1_ESM.docx]
